# Warning from a pediatric patient with severe myositis ossificans combined with femoral fracture: a case report

**DOI:** 10.3389/fped.2025.1599117

**Published:** 2025-07-14

**Authors:** Mengyao Wang, Jin Cao, Huanye Zhu

**Affiliations:** Department of Orthopedics, Ningbo No. 6 Hospital, Ningbo, Zhengjiang, China

**Keywords:** myositis ossificans, child, conservative treatment, femoral fracture, bone health

## Abstract

Myositis ossificans (MO) is a self-limiting benign ossification disorder, characterized by heterotopic ossification within skeletal muscles. Pediatric MO cases are rarer, easy to be misdiagnosed. The diagnosis of traumatic MO is based on a thorough history, physical examination, and imaging studies. Early and proactive conservative treatment can often be effective. We report a case of a 6-year-old patient with a femoral fracture combined with severe MO. He was misdiagnosed at a community hospital as having a bone tumor combined with a pathological femoral fracture and was referred to our hospital. Upon examination, we found that his condition was unusual. The patient, a child taekwondo athlete, was injured during high-intensity training, and upon examination, severe MO was found around his hip area. Ultimately, he underwent minimally invasive surgery with elastic intramedullary nails for the femoral fracture, and we adopted a conservative treatment strategy for MO. After one year of follow-up, the patient recovered well with no signs of recurrence of MO. This case report highlights the health risks faced by child athletes during high-intensity training. Repeated muscle injuries can lead to MO, and in severe cases, complications such as fractures. When diagnosing traumatic MO, it is imperative to obtain a comprehensive history of prior trauma and muscle injuries. This information can facilitate differentiation of the condition from other diseases, such as osteosarcoma, especially in the absence of histological evidence. In the case, during a one-year follow-up, the affected limb of the patient showed good functional recovery with no recurrence of MO, demonstrating the effectiveness of surgical treatment for the femoral fracture and conservative treatment for MO.

## Introduction

Myositis ossificans (MO) is a self-limiting benign ossification disorder (1), characterized by heterotopic ossification within skeletal muscles (2). Although the exact mechanism of MO remains unclear, muscle injury is considered the primary cause of traumatic MO (3). Pediatric cases of MO are particularly rare (1), easy to be misdiagnosed. The diagnosis of traumatic MO is typically based on a thorough history, physical examination, and imaging studies (4, 5). While biopsy can aid in diagnosing MO, this invasive procedure may result in further local muscle bleeding and exacerbate the progression of MO (1).

This report details a case involving a beginner taekwondo child who sustained a femoral fracture during high-intensity training. Initially diagnosed with a malignant tumor and pathological femoral fracture at a community hospital, the patient was referred to our facility. Upon further evaluation, the diagnosis was revised to severe multiple MO involving the pelvis and proximal femurs, along with a femoral fracture due to excessive training. The femoral fracture was treated using minimally invasive elastic intramedullary nails, and conservative treatment was adopted for MO. Follow-up revealed that that the femoral fracture healed well and MO gradually improved with the cessation of intense training, and no recurrence was observed.

## Case presentation

**Figure 1:**
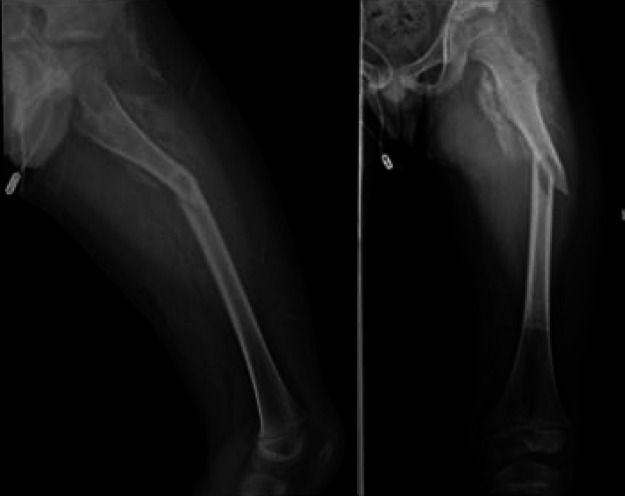
The x-ray of community hospital showed a fracture of the left proximal femur and a massive osteogenesis-like lesion of the left proximal left femur.

**Figure 2:**
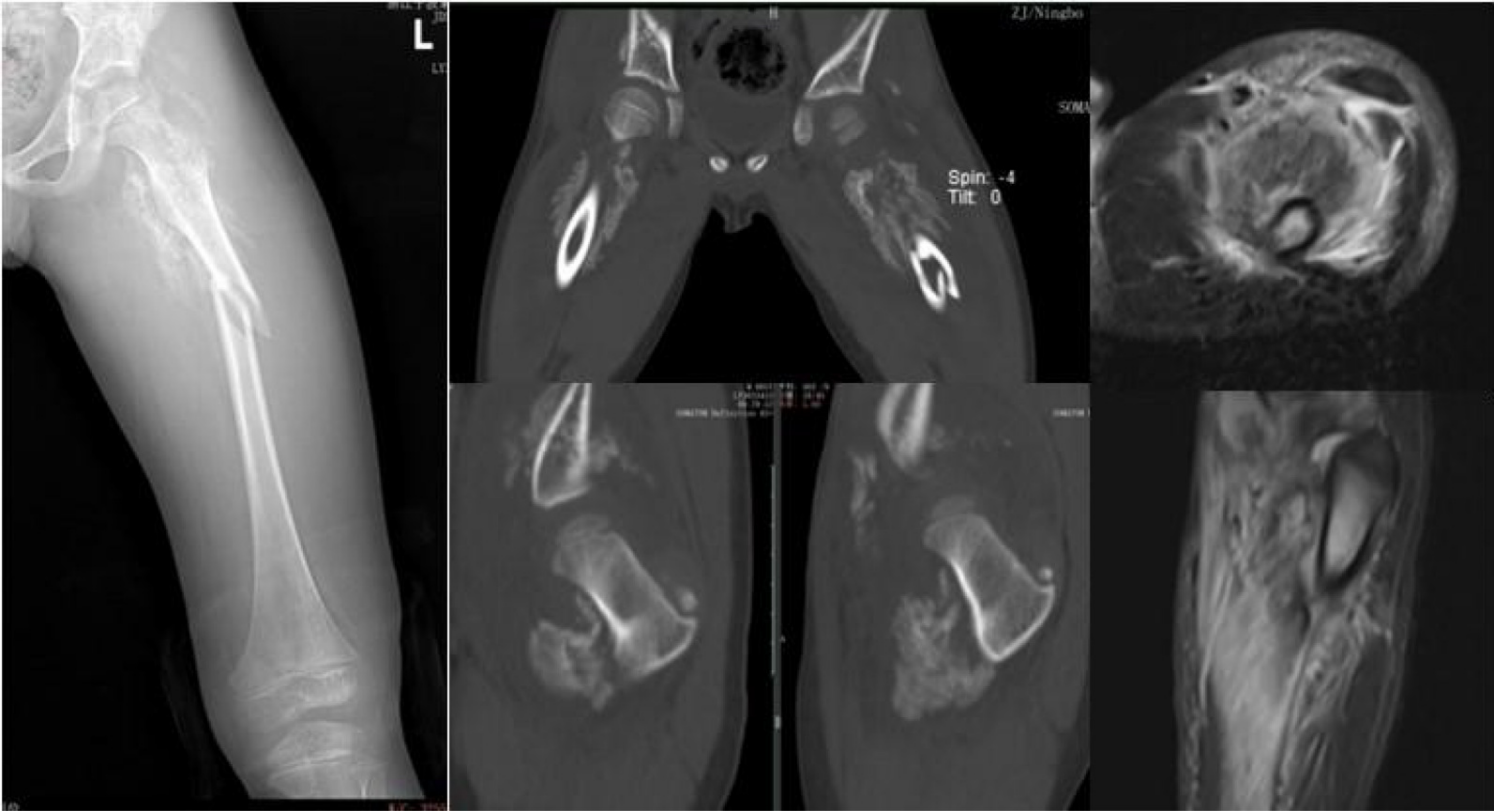
The CT showed fracture of the left upper femur segment, extensive ossification density around the gluteus, left iliac muscles and biperifemoral muscle groups, and MRI showed extensive ossification signals in the left iliac muscle and some muscle groups of both thighs, both suggesting Mo.

A male patient, six-year-old, presented to the community hospital with complaints of swelling, pain, deformity, and restricted movement in the left thigh. The initial diagnoses were “1. Pathological fracture of the left femur, 2. Suspected osteosarcoma of the proximal left femur” (Figure 1). Five hours later, the patient was referred to our hospital for further evaluation. Upon thorough history taking and examination, we discovered a more complex injury pattern. Two months prior, the patient had been enrolled in a rigorous closed taekwondo training program due to his interest in the sport. During a high-intensity training session, improper handling by the coach led to a femoral fracture. Physical examination: Extensive hard masses in both hip joints and the inner thigh of the contralateral side. Swelling in the affected limb due to the fracture, but a painless mass was palpable proximal to the fracture site. CT Revealed a fracture in the upper segment of the left femur, extensive ossification densities around the gluteal muscles bilaterally, left iliacus muscle, and surrounding muscle groups of both femurs, suggesting MO (Figure 2). MRI Showed a fracture in the upper segment of the left femur, extensive ossification signals in the left iliacus muscle and certain muscle groups of both thighs, consistent with MO (Figure 2). Based on the patient's clear history of persistent muscle injuries, physical examination, and imaging studies, we diagnosed the patient with “1. Left femoral fracture, 2. Multiple myositis ossificans”. Two days after admission, we performed minimally invasive elastic nailing for the femoral fracture. Surgical procedure: by general anesthesia, the patient was placed in a supine position. Routine sterilization and draping of the surgical field were performed. A 1 cm incision was made on both the medial and lateral sides of the distal left thigh, soft tissue was separated to expose the bone surface. Two 2.5 mm elastic nails (Double Medical Technology Inc., Xiamen, China) were inserted from the medial and lateral sides of the distal femur just above the growth plate, directed upwards towards the fracture site. Under C-arm fluoroscopy, the fracture was closed and successfully reduced. The elastic nails were advanced into the proximal fracture site. C-arm fluoroscopy confirmed proper alignment and positioning of the internal fixation.

**Figure 3:**
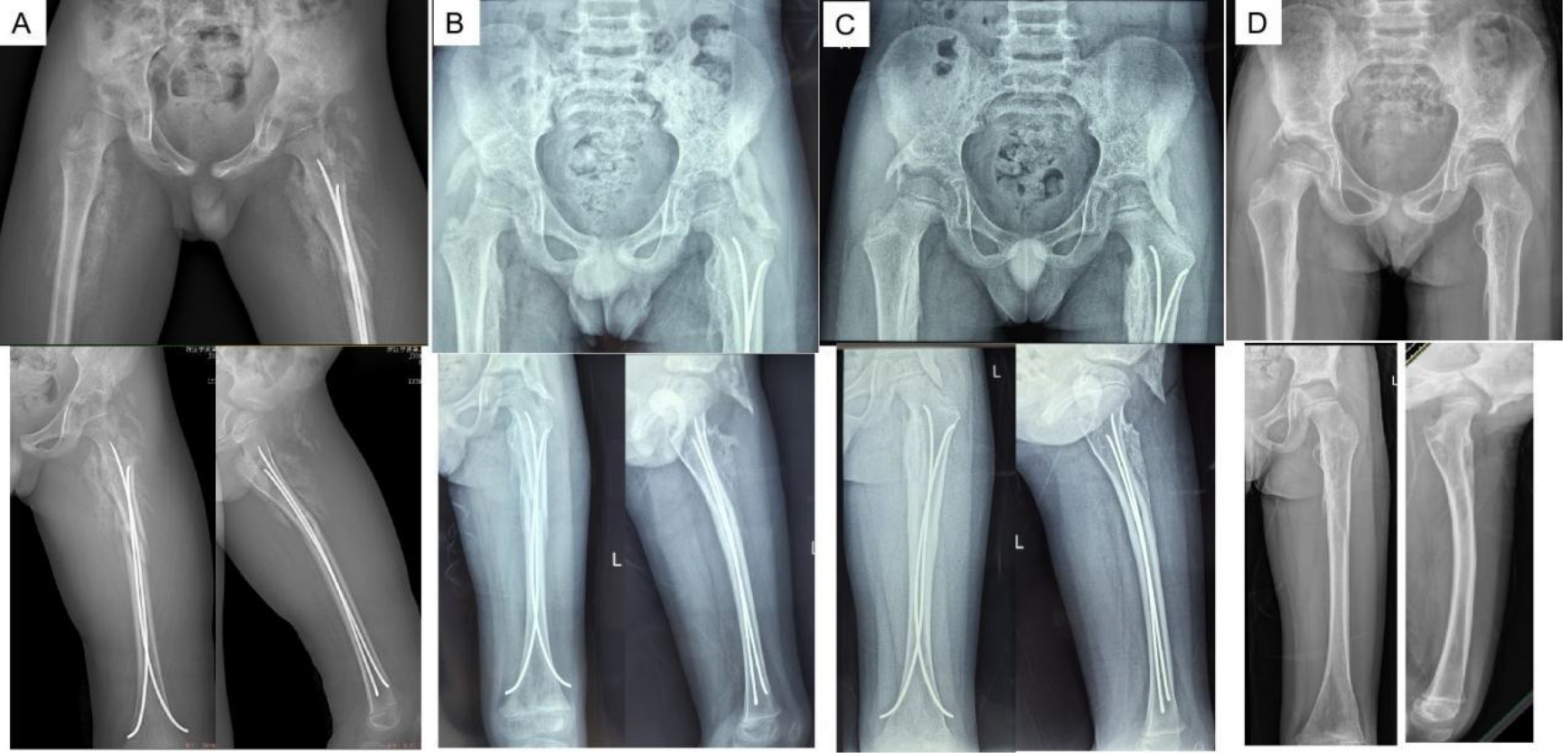
x-rays after surgery [1 month **(A)**, 3 months **(B)**, 6 months **(C)**, 12 months **(D)**] are shown that the left femoral shaft fracture was healed well and ossising myositis gradually recovered with no signs of recurrence.

**Figure 4:**
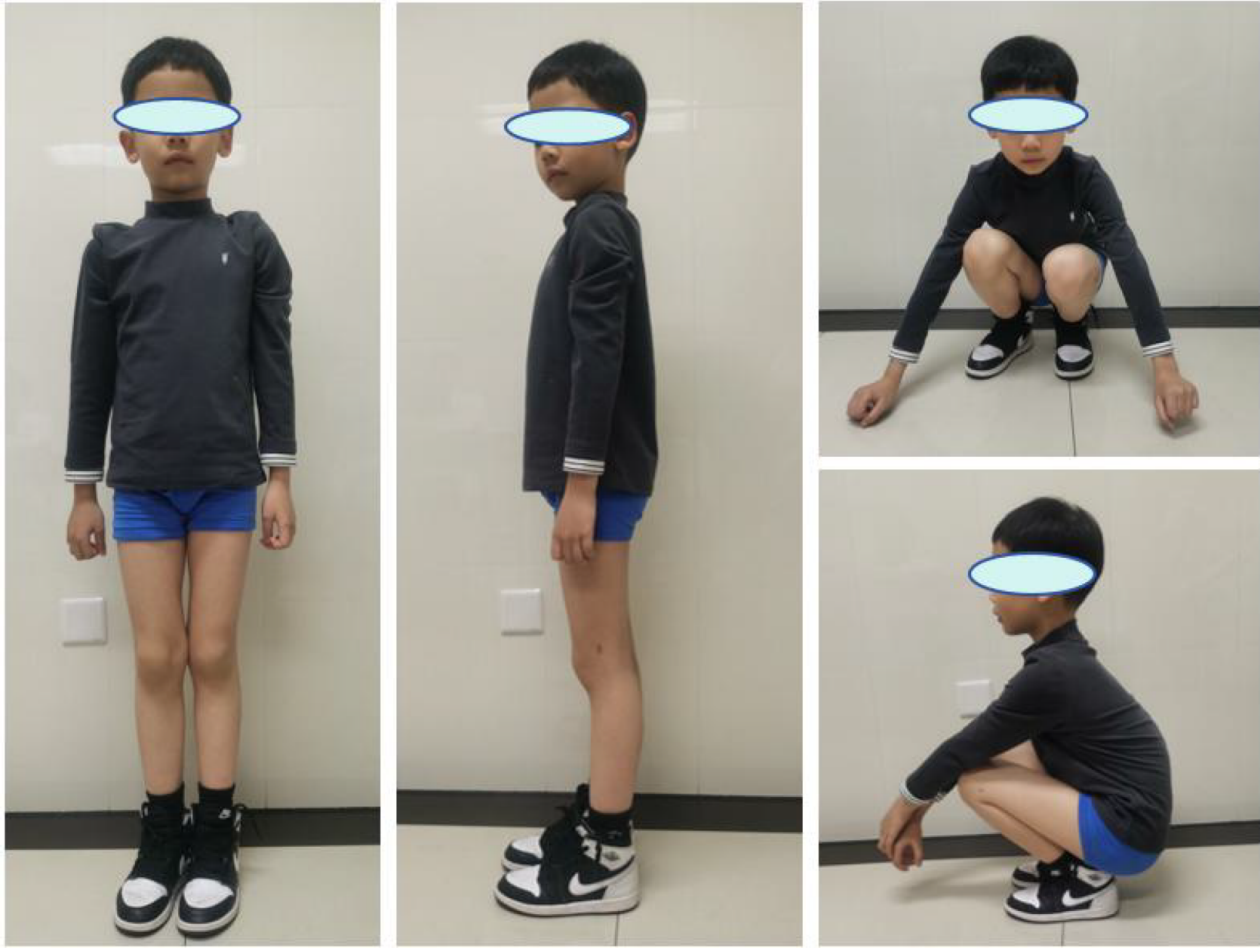
At the final follow-up (12 months), no functional impairment in the lower limb.

After the operation, parents were informed that no doctor agreed to avoid taekwondo training. The patient was followed up four times in total (1 month, 3 months, 6 months, and 1 year postoperatively) (Figure 3). Six months after surgery, the internal fixation was removed from the femur. At the final follow-up (12 months), significant improvement in the child's MO was observed, with no functional impairment in the hip joint mobility (Figure 4).

## Discussion and conclusion

MO is a non-neoplastic heterotopic ossification disorder. Although the specific etiology and pathogenesis of MO remain unclear, it is widely recognized as secondary to either traumatic or non-traumatic muscular lesions (6). Traumatic MO is thought to result from the abnormal differentiation of osteoblasts and chondrocytes during the injury and regeneration of muscle tissue, leading to the formation of cartilaginous tissue that eventually ossifies into heterotopic bone (7, 8). Histologically, early-stage MO may resemble a osteosarcoma (9, 10), and biopsy may not completely rule out malignancy. In the intermediate and late stages, a combination of patient history and imaging studies such as x-rays, computed tomography (CT), and magnetic resonance imaging (MRI) can effectively diagnose MO (11). This underscores the importance of a meticulous history, including looking for a history of trauma or causes of muscular micro-injuries (e.g., excessive activity) (7).

In this case, a 6-year-old child, was enrolled by the parents in a closed taekwondo training camp. The training regimen mainly included a significant amount of kicking, spinning, and jumping, which required frequent flexion and rotation of the hip joints, and the child described repeated episodes of pain around both hips prior to the femur fracture. Detailed medical history data, in conjunction with comprehensive physical examination and imaging findings, provide a robust foundation for revising the initial diagnosis made by community hospitals and arriving at an accurate diagnosis. Although tissue biopsy can offer definitive histological evidence, both needle and open biopsies pose significant risks of increased local trauma and may exacerbate the progression of ossifying myositis (1, 11). Consequently, we opted against performing tissue biopsies.

For the treatment of MO, early and proactive conservative treatment can often be effective (1). Surgical excision of MO is also a treatment option, particularly for cases located in the head and neck region or those causing symptomatic compression of surrounding vital structures (7). While surgical intervention can be beneficial for certain cases of MO, especially those causing functional impairment or severe symptoms, the potential for recurrence necessitates careful postoperative management and monitoring. This patient had extensive lesions, excessive surgical resection injury and high risk of recurrence, so we adopted the strategy of conservative treatment. Conservative treatment strategies for MO include rest, physical therapy, and the use of nonsteroidal anti-inflammatory drugs (NSAIDs) to alleviate pain and inflammation. After one year of follow-up, the MO in this case was clearly absorbed and there were no signs of recurrence, which demonstrated the effectiveness of conservative treatment.

The femoral fracture in this case was treated with minimally invasive elastic nailing, with the entry point at the distal femur to avoid additional damage to surrounding muscles at the fracture site, thereby preventing the exacerbation of MO. Three months after surgery, the patient recovered his normal walking function, and we removed the elastic intramedullary nail 6 months after surgery. After 1 year of follow-up, we found that the patient recovered well after surgery with no obvious signs of recurrence of MO.

This case report highlights the health risks faced by child athletes during high-intensity training. In pediatric patients, repeated muscle injuries can lead to traumatic MO, and in severe cases, it can result in fractures. When diagnosing traumatic MO, it is essential to obtain a comprehensive history of prior trauma and muscle injuries. This information can facilitate differentiation of the condition from other diseases, such as osteosarcoma, especially in the absence of histological evidence. In the case, during a one-year follow-up, the affected limb of the patient showed good functional recovery with no recurrence of MO, demonstrating the effectiveness of surgical treatment for the fracture and conservative treatment for MO.

## Data Availability

The raw data supporting the conclusions of this article will be made available by the authors, without undue reservation.
